# Subtotal cholecystectomy for Mirizzi syndrome: Should we ever remove the stone? A case report

**DOI:** 10.1016/j.amsu.2022.103381

**Published:** 2022-02-12

**Authors:** Michela Zanatta, Giovanna Brancato, Guido Basile, Francesco Basile, Marcello Donati

**Affiliations:** a Surgical Clinic Unit, Department of Surgery and Medical-Surgical Specialties. University of Catania, 95123, Catania, Italy; b Emergency and Abdominal Surgery Unit, Department of Surgery and Medical-Surgical Specialties. University of Catania, 95123, Catania, Italy

**Keywords:** Mirizzi syndrome, Subtotal cholecystectomy, Hepatic abscess, Intra-hepatic gallbladder perforation, Open cholecystectomy, Case report, WBC, white blood cells, US, Ultrasound, CT scan, Computed Tomography scan

## Abstract

**Introduction and importance:**

Mirizzi Syndrome is a rare complication of cholelithiasis.

In this case report the Authors present an original surgical approach for the treatment of complicated gallbladders, based on open subtotal cholecystectomy, leaving in situ the stone. This is the first case showing safety and reliability of the present strategy at a four-year follow-up.

**Case presentation:**

A 68-year-old patient came to our emergency room with abdominal pain, leukocytosis and fever. At surgical exploration he presented a sclerotic retraction of the gallbladder together with an intrahepatic abscess, that forced us first to perform an open subtotal cholecystectomy, resecting the gallbladder cranially and leaving in situ the stone.

**Clinical discussion:**

The post-operative course was uneventful. The four-year clinical, US and CT scan follow-up was negative and the patient referred a normal quality of life. The present strategy could be considered an intraoperative rescue option in such a complex operative scenario in which is impossible to safely remove the stone.

**Conclusion:**

This case report demonstrates how in selected cases, when absolutely necessary and unavoidable without high risks, the stone can be left in situ as an eventual stone resection would be extremely risky.

## Highlights

• Subtotal cholecystectomy is a well-known and established procedure in case of difficult gallbladders as in Mirizzi syndrome.
• A traditionally accepted part of this technique is to remove the stone together with the gallbladder.
• In selected cases the removal of the stone could be difficult and even dangerous for the patient.
• Necessity of stone removal in difficult gallbladders should be carefully counterbalanced with possible local complications.
• Four-year follow-up without complications is showing that in selected cases the stone can be left in situ.

## 1 Introduction

This case report has been reported in line with the SCARE Criteria [1].

Mirizzi syndrome, also known as extrinsic bile compression syndrome, is a complication of cholelithiasis, primarily described by Kehr [2] and Ruge [3] in the early 1900s and only named after Mirizzi in 1948 [4,5]. Many classifications have been made for Mirizzi syndrome: the most accepted one is the classification made by McSherry et al., in 1982 [6]. A milestone of the surgical treatment of cholelithiasis is the removal of the gallbladder together with its contents (the stones) in order to prevent surgical complications and well-known evolutions of the disease. Here we report a case of Mirizzi syndrome complicated by a liver abscess, and its successful original treatment strategy. The work has been reported in line with the SCARE 2020 criteria [7].

## 2 Presentation of case

**Fig. 1:**
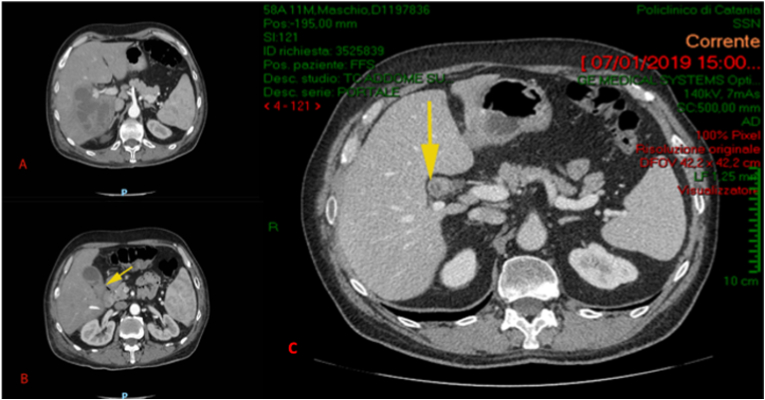
Pre-operative and follow-up CT scan. 1A. Giant hepatic abscess due to intrahepatic gallbladder perforation. 1B. Stone in the cystic stump (yellow arrow) in Mirizzi syndrome. 1C. Follow-up: after four years the stone is in-situ (yellow arrow), retracted in the hepatic hilum, without any sign of inflammation. . (For interpretation of the references to colour in this figure legend, the reader is referred to the Web version of this article.)

A 67-year-old patient came to our clinical observation in emergency room: male patient, married, truck driver, affected by type II diabetes in good compensation by Metformin assumption, under oral anti-coagulation due to stent positioning for a previous heart attack. He came with a 40 °C intermittent fewer from 2 days, extreme leukocytosis (30.000 WBC) and acute abdominal pain at the right hypochondrium. Murphy's sign manually evocated was positive. No family history of biliary disease was reported. A CT scan of the abdomen revealed a giant hepatic abscess involving the entire right lobe till the falciform ligamentum (Fig. 1A) and a hydropic gallbladder with a big stone incarcerated into the cystic duct described as very short (Fig. 1B). The abscess was drained by the interventional radiologists of our Hospital two times in 4 days and showed complete regression within 5 days, as well as the leukocytosis and temperature. All laboratory parameters reverted to normal and the procedure was well-tolerated by the patient. After 3 weeks of antibiotic therapies and US controls, in order to prevent recurrences, we decided to surgically treat the hydropic gallbladder that was the *primum movens* of the intrahepatic perforation and caused the abscess. The operation was performed by the last Author of the present report as an open procedure. The intraoperative findings showed a complex retraction of the gallbladder hilar portion with duodenal retraction and rotation inside the liver hilar portion.

## 3 Results

**Fig. 2:**
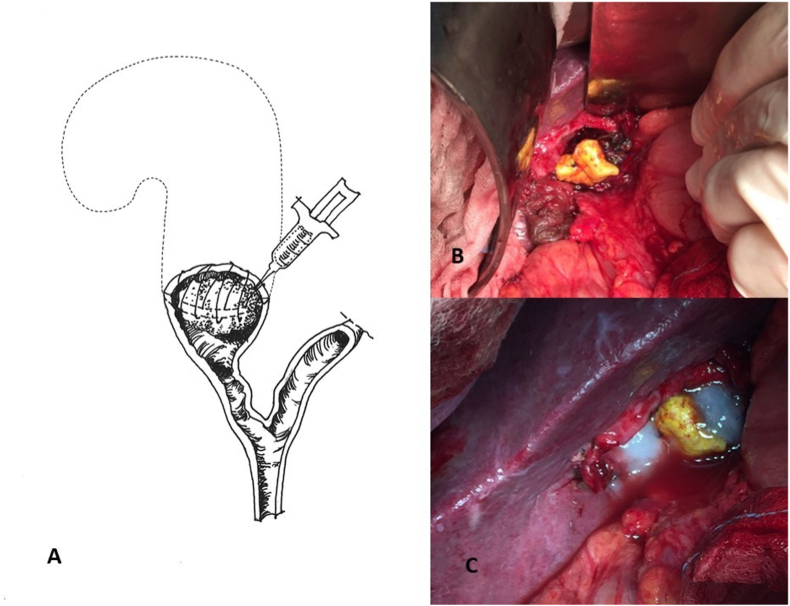
Clinical pictures. 2A. Schematic illustration of the operation. 2B. Operative field after subtotal cholecystectomy and positioning of TachoSil®. 2C. Operative field after the apposition of fibrin glue.

Given the sclerotic retraction of the gallbladder, we performed the subtotal cholecystectomy, opening the gallbladder: the digital exploration of the hilar part guided the gallbladder resection. Due to the incarceration of the stone inside the cystic stump (Fig. 2) and the very short cystic duct (Mirizzi Syndrome Type I), so like the rotation of the hilar elements and the intimate contact between the cystic stump and the right portal branch showed at the preoperative CT scan, and given the high risk of intraoperative lesions of the common bile duct and of the portal vein, we decided to leave the stone inside the cystic stump, folding the extremities of the gallbladder infundibular portion. This way, we covered the stone “gluing” it by applying fibrin glue over TachoSil® Fibrin Sealant (Fig. 2). The postoperative course was completely uneventful. The patient was discharged 4 days after the operation with free oral intake and no drugs assumption. The US and CT scan follow-up (Fig. 1C), after four years, showed the “stone” buried inside the residual cystic “stump”. Nowadays, the patient is asymptomatic, no recurrences were observed, liver function test are normal. The patient reported a good quality of life referring complete absence of symptoms during four years of follow-up.

## 4 Discussion

The Gold Standard treatment of non-complicated cholelithiasis is laparoscopic cholecystectomy [8]. However, there are still today some clinical conditions (difficult gallbladders) that may require an open approach d'embleè, given the reported very high percentage of conversion to open surgery [9]: among those there is Mirizzi syndrome, due to the intense degree of fibrosis and difficult anatomy [10]. According to the classification made by McSherry et al. [6], Mirizzi syndrome recognizes two different patterns: Type I when there is only the extrinsic compression of the common bile duct and Type II when there is a concomitant cholecystobiliary fistula. Laparoscopic approach is commonly accepted for Mirizzi Syndrome Type I at early stage, but sometimes an open approach is preferred due to the intense inflammation and the severe fibrosis, which usually requires a subtotal cholecystectomy [8].

In our case, not only the intense fibrosis and retraction of the gallbladder, but even the previous drainage of intrahepatic abscess due to intrahepatic gallbladder perforation, forced us to prefer an open approach [9,11]. Out of that, it was necessary to perform a subtotal cholecystectomy leaving “in situ” the gallbladder infundibular part. This approach is accepted and codified especially in emergency conditions [12]. Given that the stone was completely cemented to the posterior wall of the infundibular part and that the hilar elements were completely retracted and rotated, out of traditional accepted approach, we left in place even the big infundibular stone, because the removal was quite impossible without risking a major lesion of common bile duct or even of the portal trunk. A further novelty in our surgical approach is the use of a hemostatic patch (TachoSil® Fibrin Sealant) to straighten the gallbladder suture, in order to avoid biliary fistulas, assuming that the use of hemostatic patches is described in the literature to protect intestinal or pancreatic-jejunal anastomosis [13]. Resecting the gallbladder cranially to the stone will be the “insurance” against future pain attacks and cholecystitis. The stone will remain buried on a granuloma as we demonstrated in our instrumental follow-up (Fig. 1C). We completed 4 years of follow-up without any abdominal pain or colic attack. The stone remained in situ without any consequence.

## 5 Conclusions

To best of our knowledge this is the first reported case of successful leave-in-situ of the stone after a subtotal cholecystectomy as a definitive treatment of Mirizzi Syndrome with long uneventful follow-up out of recommendations or guidelines. Given that the prior goal of the treatment is to remove gallbladder and stones, we showed that in selected cases, when absolutely necessary and unavoidable without high risks, the stone can be left in situ as an eventual stone resection would be extremely risky.

## 6 Consent of patient

Written informed consent was obtained from the patient for publication of this case report and accompanying images. A copy of the written consent is available for review by the Editor-in-Chief of this journal on request.

## Sources of funding for your research

No sources of funding declared.

## Ethical approval

No ethical approval needed for this study.

## Consent

Written informed consent was obtained from the patient for publication of this case report and accompanying images. A copy of the written consent is available for review by the Editor-in-Chief of this journal on request.

## Author contribution

MZ wrote the draft of the work, collected clinical data, made follow-up and literature search; GB1 collected images and wrote comments; GB2 made interpretation of data, FB made literature search and first review; MD made study design and final revision. MD and FB performed the operation. Each Author approved the submitted version.

## Registration of research studies

Not appliable

## Guarantor

Prof. Marcello Donati.

## Conflicts of interest

The Authors declare they have no conflict of interest.

## References

[1] Agha R.A., Franchi T., Sohrabi C., Mathew G., Kerwan A., SCARE Group (2020 Dec). The SCARE 2020 guideline: updating consensus surgical CAse REport (SCARE) guidelines. Int. J. Surg..

[2] Kehr H. (1905).

[3] Ruge E. (1908). Beiträge zur chirurgischen Anatomie der grossen Gallenwege. Arch. f. klin. Chir..

[4] Mirizzi P.L. (1948). Síndrome del conducto hepático. J. Int. Chir..

[5] Acquafresca P., Palermo M., Blanco L., García R., Tarsitano F. (2014). Síndrome de Mirizzi: prevalencia, diagnóstico y tratamiento. Acta Gastroenterol. Latinoam..

[6] McSherry C.K. (1982). The Mirizzi syndrome: suggested classification and surgical therapy. Surg. Gastroenterol..

[7] Agha R.A., Franchi T., Sohrabi C., Mathew G., for the SCARE Group (2020). The SCARE 2020 guideline: updating consensus surgical CAse REport (SCARE) guidelines. Int. J. Surg..

[8] van Dijk A., de Reuver P., Besselink M. (2017). Assessment of available evidence in the management of gallbladder and bile duct stones: a systematic review of international guidelines. HPB.

[9] Sciumè C., Geraci G., Li Volsi F., Pisello F., Modica G. (2002). Atypical presentation of a case of Mirizzi syndrome simulating cholangiocarcinoma. Ann. Ital. Chir..

[10] Ashfaq A., Ahmadieh K., Shah A.A. (2016). The difficult gall bladder: outcomes following laparoscopic cholecystectomy and the need for open conversion. Am. J. Surg..

[11] Donati M., Biondi A., Basile F., Gruttadauria S. (2014). An atypical presentation of intrahepatic perforated cholecystitis: a modern indication to open cholecystectomy. Report of a case. BMC Surg..

[12] Elshaer M., Gravante G., Thomas K. (2015). Subtotal cholecystectomy for "difficult gallbladders": systematic review and meta-analysis. JAMA Surg..

[13] Lewis K.M., Ikeme S., Olubunmi T., Kuntze C.E. (2018). Clinical effectiveness and versatility of a sealing hemostatic patch (HEMOPATCH) in multiple surgical specialties. Expet Rev. Med. Dev..

